# Supplementary material for: Green Graphene–Chitosan Sorbent Materials for Mercury Water Remediation
Source: Nanomaterials (Basel). 2020 Jul 28;10(8):1474. doi: 10.3390/nano10081474 (PMC7466593; doi:10.3390/nano10081474)
Supplement: Supplementary file 1 [file nanomaterials-10-01474-s001.pdf]

Supplementary Materials

Green Graphene–Chitosan Sorbent Materials for Mercury Water Remediation

Ana Bessa [1,2], Gil Gonçalves [1], Bruno Henriques [2,3], Eddy M. Domingues [1], Eduarda Pereira [3] and Paula A. A. P. Marques [1*]

[1] Centro de Tecnologia Mecânica e Automação (TEMA), Mechanical Engineering Department, University of Aveiro, 3810-193 Aveiro, Portugal; arcb@ua.pt (A.B.); ggoncalves@ua.pt (G.G.); eddy@ua.pt (E.M.D.)

[2] Centro de Estudos do Ambiente e do Mar (CESAM) & Department of Chemistry, University of Aveiro, 3810-193 Aveiro, Portugal; brunogalinho@ua.pt

[3] Laboratório Associado para a Química Verde-Rede de Química e Tecnologia (LAQV-REQUIMTE) & Department of Chemistry, University of Aveiro, 3810-193 Aveiro, Portugal; eduper@ua.pt

* Correspondence: paulam@ua.pt

BET analysis of GO–CH aerogel

The result for the specific surface area (S_{BET}) of the GO–CH aerogel was very low (9.06 m^2 g^{-1}, with a pore volume of 0.016 cm^3 g^{-1}). This is not uncommon, as the S_{BET} of graphene-based aerogels is normally substantially lower than the expected value for the single-sheet graphene (~2600 m^2 g^{-1}) [1]. The determination of S_{BET} for GO-based aerogels is generally quite challenging and is normally largely underestimated. The possible overlapping of graphene sheets, high degree of oxidation and the presence of water molecules at the surface may difficult the access of N_2 molecules, thus triggering such low S_{BET} values.[2] In a previous study of GO aerogels, we observed H3-type hysteresis loop, which can be assigned to the presence of plate-like particle aggregates and slit-shaped pores. The plateau was not reached even in saturation conditions, which can be a result of unrestricted multilayer adsorption in large mesopores and macropores, which can ultimately hinder an accurate estimation of the real pore volume [3].

Table S1. Sorption reaction kinetic models.

Kinetic model	Equation	References
Pseudo-first-order (Lagergren)	$q_t = q_e\left(1 - e^{-k_1 t}\right)$	[4]
Adsorption capacity Pseudo-second-order (Ho)	$q_t = \frac{q_e^2 k_2 t}{1 + q_e k_2 t}$	[5]
Elovich	$q_t = \frac{1}{\beta}\ln(1 + \alpha\beta t)$	[6]

q_t is the amount of metal sorbed per gram of sorbent at time t (μmol g^{-1}), q_e amount of metal adsorbed per gram of sorbent at equilibrium (μmol g^{-1}), k_1 rate constant of pseudo-first order (h^{-1}), k_2 rate constant of pseudo-second order (g μmol^{-1} h^{-1}), α initial sorption rate (μmol g^{-1} h^{-1}), β desorption constant (g μmol^{-1}).

In addition to the reaction models, two widely known diffusion-based models, Boyd's film-diffusion [7] and Webber's pore-diffusion [8], were used in order to analyse the sorption mechanism and the rate-controlling step involved in the sorption process.

Boyd's film-diffusion model presumes that the main resistance to diffusion is in the boundary layer surrounding the adsorbent particle[9,10] and it is expressed as:

$$F = 1 - \frac{6}{\pi^2} \sum_{n=1}^{\infty} \left(\frac{1}{n^2} \right) \exp.\left(-n^2 Bt \right) \quad (4)$$

where F is the fractional attainment of equilibrium, at different times, t, and Bt is a function of F:

$$F = \frac{q_t}{q_e} \quad (5)$$

Bt can be calculated as:

For F values > 0.85, $$Bt = -0.4977 - \ln\left(1 - F\right) \quad (6)$$

For F values < 0.85, $$Bt = \left(\sqrt{\pi} - \sqrt{\pi - \frac{\pi^2 F}{3}} \right)^2 \quad (7)$$

If the plot Bt vs t (Boyd's plot) excludes the origin, on can conclude that film diffusion or chemical reaction is the rate-controlling step. If the plot is linear and passes through the origin the intra-particle diffusion controls the rate of mass transfer.

Weber's intraparticle-diffusion model is defined by the following equation [9,10]:

$$q_t = k_i t^{1/2} \quad (8)$$

Where k_i is the intraparticle-diffusion parameter (mg g^{-1} $h^{-1/2}$). If intraparticle-diffusion is the rate-limiting step, then a plot of qt vs t will give a straight line with a slope that equals ki and an intercept equal to zero. If not, some other mechanism along with intraparticle diffusion must also be involved. The analysis of the experimental data using film-diffusion and the intraparticle-diffusion models, as well as the prediction of the diffusion coefficients, was performed following the piecewise linear regression methodology (PLR) proposed by Malash et al. [9] using a Microsoft® Excel™ worksheet developed by these authors.

Table S2. Elemental analysis of natural waters before and after Hg removal studies by GO–CH.

**Water source**	**Tap**		**River σ = 33.1 μS/m**		**Sea Sal. = 34.7**	
	Initial	GO–CH	Initial	GO–CH	Initial	GO–CH
pH	7.13	7.45	4.30	4.32	7.80	7.69
Major elements (mg L^{-1})						
Ca	33	33	1.3	1.3	411	399
Na	8.5	8.3	5.5	4.9	>1000	>1000
K	1.9	1.9	0.5	0.5	>500	>500
Mg	2.0	2.0	0.5	0.5	>2000	>2000
Minor elements(μ L^{-1})						
B	10	7.5	4.3	4.3	5346	5340
Al	34	<5	54	54	178	86
Cr	0.7	<0.5	<0.5	<0.5	16.8	16.8
Fe	54	<10	12	<5	351	351
Co	<0.1	<0.1	<0.1	<0.2	<40	<40
Ni	<1	<1	<1	<1	<20	<20
Cu	2.7	<1	<1	<1	<20	<20
Zn	76	31	7.8	5	1120	1000
As	<2	<2	<2	<2	<100	<100
Se	<2	<2	<2	<2	185	185
Sr	21	21	7	7	9338	9338

Cd	<0.1	<0.1	0.21	<0.1	<40	<40
Sb	<0.1	<0.1	<0.1	<0.1	<2	<2
Ba	6.1	5.3	2.3	2.0	160	158
Pb	<0.1	<0.1	2.2	<0.2	<80	<80

References

1. Rao, C.N.R.; Sood, A.K.; Subrahmanyam, K.S.; Govindaraj, A. Graphene: The new two-dimensional nanomaterial. *Angew. Chemie - Int. Ed.* **2009**, *48*, 7752–7777, doi:10.1002/anie.200901678.
2. Worsley, M.A.; Pauzauskie, P.J.; Olson, T.Y.; Biener, J.; Satcher, J.H.; Baumann, T.F. Synthesis of graphene aerogel with high electrical conductivity. *J. Am. Chem. Soc.* **2010**, *132*, 14067–14069, doi:10.1021/ja1072299.
3. Borrás, A.; Gonçalves, G.; Marbán, G.; Sandoval, S.; Pinto, S.; Marques, P.A.A.P.; Fraile, J.; Tobias, G.; López-Periago, A.M.; Domingo, C. Preparation and Characterization of Graphene Oxide Aerogels: Exploring the Limits of Supercritical CO2 Fabrication Methods. *Chem. - A Eur. J.* **2018**, *24*, 15903–15911, doi:10.1002/chem.201803368.
4. Lagergren, S. About the theory of so-called adsorption of soluble substances. *K. Sven Veten Hand* **1898**, *24*, 1–39.
5. Ho, Y.S.; McKay, G. Pseudo-second order model for sorption processes. *Process Biochem.* **1999**, *34*, 451–465, doi:Doi 10.1016/S0032-9592(98)00112-5.
6. Low, M.J.D. Kinetics of Chemisorption of Gases on Solids. *Chem. Rev.* **1960**, *60*, 267–312, doi:Doi 10.1021/Cr60205a003.
7. Boyd, G.E.; Adamson, A.W.; Myers, L.S. The Exchange Adsorption of Ions from Aqueous Solutions by Organic Zeolites .2. *J. Am. Chem. Soc.* **1947**, *69*, 2836–2848, doi:Doi 10.1021/Ja01203a066.
8. Weber, W.J.; Morris, J.C. Kinetics of adsorption on carbon from solution. *Kinet. Adsorpt. Carbon from Solut.* **1963**.
9. Malash, G.F.; El-Khaiary, M.I. Piecewise linear regression: A statistical method for the analysis of experimental adsorption data by the intraparticle-diffusion models. *Chem. Eng. J.* **2010**, *163*, 256–263, doi:10.1016/j.cej.2010.07.059.
10. Ho, Y.S.; Ng, J.C.Y.; McKay, G. Kinetics of pollutant sorption by biosorbents: Review. *Sep. Purif. Methods* **2000**, *29*, 189–232, doi:10.1081/SPM-100100009.
